# Reply to Karademir, F.; Fírat, T. Comment on “Covelli et al. Extracorporeal Shock Wave Therapy (ESWT) vs. Exercise in Thumb Osteoarthritis (SWEX-TO): Prospective Clinical Trial at 6 Months. *Life* 2024, *14*, 1453”

**DOI:** 10.3390/life15030385

**Published:** 2025-02-28

**Authors:** Ilaria Covelli, Silvana De Giorgi, Antonio Di Lorenzo, Angelo Pavone, Fabrizio Salvato, Francesco Rifino, Biagio Moretti, Giuseppe Solarino, Angela Notarnicola

**Affiliations:** 1 Orthopedics Unit, Department of Translational Biomedicine and Neuroscience “DiBraiN”, School of Medicine and Surgery, University of Bari, General Hospital, 70124 Bari, Italy; ilaria.covelli@uniba.it (I.C.); silvana.degiorgi@uniba.it (S.D.G.); a.pavone20@studenti.uniba.it (A.P.); f.salvato1@studenti.uniba.it (F.S.); francesco.rifino@policlinico.ba.it (F.R.); biagio.moretti@uniba.it (B.M.); giuseppe.solarino@uniba.it (G.S.); 2 Interdisciplinary Department of Medicine, University of Study of Bari, Piazza Giulio Cesare 11, 70124 Bari, Italy; antonio.dilorenzo@uniba.it

Dear Drs. Karademir and Fírat, we thank you for your Letter [1] in response to our article “Extracorporeal Shock Wave Therapy (ESWT) vs. Exercise in Thumb Osteoarthritis (SWEX-TO): Prospective Clinical Trial at 6 Months” [2].

As far as statistical analysis is concerned, we chose to employ the Wilcoxon rank-sum test for two main reasons. First, only two groups (exercise-only and Extracorporeal Shock Wave Therapy) were being confronted, making it possible to use a mean confrontation method. Secondly, the non-normal distribution of study variables made us prefer a non-parametric method, which was more consistent with the measures from a mathematical standpoint. In addition to this, we specify that the Friedman test was also applicable to our study, but was deemed too conservative. Our investigation was, in fact, designed with an exploratory aim. Further investigation could use this different line of testing.

Regression analysis was used to confront the two therapies by translating the “therapy” variable into a binomial one. By doing so, we were able to observe how the introduction of Extracorporeal Shock Wave Therapy interacted with our dependent variables both in combination with other independent variables (in multilinear models) and alone (in unilinear regression). We agree that different kinds of analysis would have been possible, and that linear regression alone is a very simple way of describing association; however, more complicated models tend to be very conservative in small samples such as ours. For future development, larger samples indeed could be studied with more refined mathematical tools.

The shock wave device (Minilith, Storz, Swiss) used in the study is equipped with an online ultrasound probe, which allowed for the optimization of the aiming and monitoring of correct positioning throughout the therapy. The device coupling interface was larger than the treatment area; therefore, ultrasound supervision allowed for the optimization of the identification of the treatment site (in this case, the trapeziometacarpal joint), also in relation to the treatment depth.

In the works relating to the infiltrative procedure of the trapeziometacarpal joint, it emerged that in the absence of instrumental control, incorrect positioning of the injection occurred in 42% of cases [3]. Therefore, like these previous experiences, in our study, the use of ultrasound control, provided in the device used, guaranteed us accurate treatment. This does not exclude that with different shock wave devices (not equipped with online ultrasound guidance), a doctor can make use of his or her own clinical experience to carry out the shock wave treatment of thumb osteoarthritis by identifying the district with the palpatory method.

The interest expressed in your letter on the effects of shock waves on osteoarthritis is understandable. Shock wave treatment, inducing a cavitation effect, triggers the repair processes of the treated tissues through the release of growth factors, modulation of phlogogenic cytokines, chemotaxis of mesenchymal stem cells, and induction of cellular proliferation and differentiation. So far, shock wave treatment in the orthopedic field has found application in the pathologies of tendon tissue (calcific and non-calcific tendinopathy) and bone (delayed consolidation of fractures, bone edema) [4].

As regards the pathology of cartilaginous tissue, we currently have support from pre-clinical research on chondrocytes, which demonstrates a modulation effect of the cytokines involved in osteoarthritic pathology [5] and the first clinical experiences, which support functional improvement and pain remission, mainly in knee osteoarthritis [6].

For now, shock wave treatment in osteoarthritic pathology falls within the experimental indications in the hands of experts. At this point, considering the potential of therapy on the pathological model of osteoarthritis, further clinical studies on humans will be necessary, which include among their end points the instrumental monitoring (with radiography, MRI, arthroscopy, etc.) of cartilaginous degeneration, formation of osteophytes and possible modification of the joint space.

Our goal is to continue scientific research to continue to introduce more information on the relevance of shock wave therapy in musculoskeletal pathologies, including osteoarthritic pathology, to provide adequate scientific evidence on treatment protocols and therapeutic appropriateness.

## Data Availability

The datasets generated and/or analyzed during the current study are available from the corresponding author upon reasonable request due to privacy.
